# Chlorido[1-(4,5-dihydro-1,3-thia­zol-2-yl-κ*N*)ethanone thio­semicarbazonato-κ^2^ *N* ^1^,*S*]nickel(II)

**DOI:** 10.1107/S1600536810051500

**Published:** 2010-12-15

**Authors:** Emilio Viñuelas-Zahínos, Francisco Luna-Giles, Pablo Torres-García, Álvaro Bernalte-García

**Affiliations:** a Departamento de Quimica Organica e Inorganica, Facultad de Ciencias, Universidad de Extremadura, Badajoz, Spain

## Abstract

In the title compound, [Ni(C_6_H_9_N_4_S_2_)Cl], the Ni atom is in a slightly distorted square-planar environment coordinated by a Cl atom and a deprotonated thio­semicarbazone ligand *via* its thia­zoline N, azomethine N and thiol­ate S atoms. Short inter­molecular N—H⋯Cl and C—H⋯S contacts are present in the crystal structure.

## Related literature

For the structure of the organic ligand and several metal complexes, see: Viñuelas-Zahínos *et al.* (2011) ▶. For the structures of closely related nickel complexes, see: Liu *et al.* (1999 ▶); Philip *et al.* (2004 ▶); Swearingen *et al.* (2002 ▶).
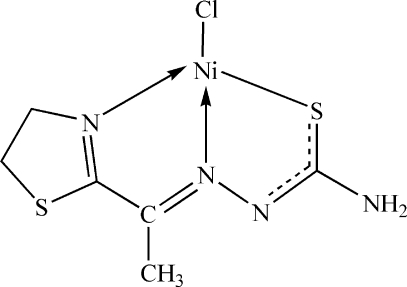


## Experimental

### 

#### Crystal data

[Ni(C_6_H_9_N_4_S_2_)Cl]
*M* *_r_* = 295.45
Monoclinic, 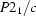

*a* = 9.656 (2) Å
*b* = 10.617 (2) Å
*c* = 11.187 (3) Å
β = 112.874 (4)°
*V* = 1056.7 (4) Å^3^
*Z* = 4
Mo *K*α radiation
μ = 2.45 mm^−1^
*T* = 298 K
0.22 × 0.15 × 0.08 mm

#### Data collection

Bruker SMART 1000 CCD diffractometer
Absorption correction: multi-scan (*SADABS*; Sheldrick, 2004 ▶) *T*_min_ = 0.615, *T*_max_ = 0.828
2554 measured reflections
2554 independent reflections
1758 reflections with 2σ(*I*)
*R*_int_ = 0.030

#### Refinement

*R*[*F* ^2^ > 2σ(*F* ^2^)] = 0.034
*wR*(*F* ^2^) = 0.076
*S* = 1.05
2554 reflections
128 parameters
H-atom parameters constrained
Δρ_max_ = 0.33 e Å^−3^
Δρ_min_ = −0.34 e Å^−3^

### 

Data collection: *SMART* (Bruker, 2001 ▶); cell refinement: *SAINT* (Bruker, 2001 ▶); data reduction: *SAINT*; program(s) used to solve structure: *SHELXS97* (Sheldrick, 2008 ▶); program(s) used to refine structure: *SHELXL97* (Sheldrick, 2008 ▶); molecular graphics: *ORTEP-3* (Farrugia, 1997 ▶); software used to prepare material for publication: *publCIF* (Westrip, 2010 ▶).

## Supplementary Material

Crystal structure: contains datablocks I, global. DOI: 10.1107/S1600536810051500/rk2251sup1.cif

Structure factors: contains datablocks I. DOI: 10.1107/S1600536810051500/rk2251Isup2.hkl

Additional supplementary materials:  crystallographic information; 3D view; checkCIF report

## Figures and Tables

**Table 1:** Hydrogen-bond geometry (Å, °)

*D*—H⋯*A*	*D*—H	H⋯*A*	*D*⋯*A*	*D*—H⋯*A*
N4—H4*B*⋯Cl^i^	0.86	2.51	3.310 (3)	155
N4—H4*A*⋯Cl^ii^	0.86	2.53	3.373 (3)	166
C3—H3*B*⋯S1^iii^	0.97	2.98	3.537 (3)	118

Symmetry codes: (i) 
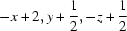
; (ii) 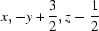; (iii) 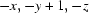.

**Fig. 1.**
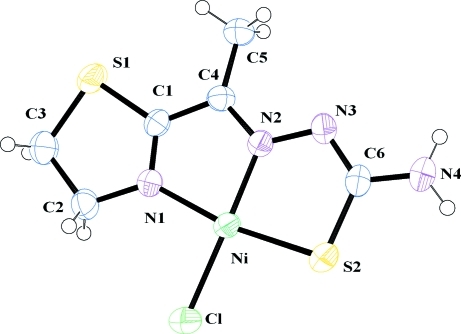
A view of molecular structure of title compound, showing the atom–numbering scheme. Displacement ellipsoids are drawn at 50% probability level. H atoms are presented as a small spheres of arbitrary radius.

## supplementary crystallographic information

### Comment

The preceding study reports the metal complexes of 1–(4,5–dihydro–1,3–thiazol–2–yl)ethanone thiosemicarbazone (HATtsc) (Viñuelas–Zahínos *et al.*, 2011). It should be pointed out that in nickel complex the organic ligand is deprotonated and shifts from the thione to the thiolate form, in such a way that the negative charge is delocalized between the two bonds S2—C6 and N3—C6, as it is observed in other nickel complexes with thiosemicarbazone ligands (Liu *et al.*, 1999; Philip *et al.*, 2004; Swearingen *et al.*, 2002). Another difference in complex with respect to the structure of the free ligand HATtsc is the degree of rotation around the C1—C4 and C6—N3 bonds, which permits coordination through N1 and S2. In crystal structure there are the following short intermolecular contacts: N4—H4A···Cl, N4—H4B···Cl and C3—H3B···S1.

### Experimental

1-(4,5-dihydro-1,3-thiazol-2-yl)ethanone thiosemicarbazone (HATtsc) was synthesized as according to a literature procedure (Viñuelas-Zahínos *et al.*, 2010). A solution containing NiCl_2_^.^6H_2_O (58.5 mg, 0.25 mmol) in 1 ml ethanol:acetonitrile (2:1) was added to a solution (40 ml) of HATtsc (50.0 mg, 0.25 mmol) in ethanol:acetonitrile (2:1). The brown product was recrystallized from ethanol:methanol (1:1) to give brown crystals.

### Refinement

All hydrogen atoms attached to carbon and nitrogen atoms were positioned geometrically and refined as riding, with C—H = 0.96–0.97Å, N—H = 0.86Å and *U*_iso_(H) = 1.2(1.5 for methyl group) *U*_eq_(C,N).

### Crystal data

**Table:**

[Ni(C_6_H_9_N_4_S_2_)Cl]	*F*(000) = 600
*M**_r_* = 295.45	*D*_x_ = 1.857 Mg m^−^^3^
Monoclinic, *P*2_1_/*c*	Mo *K*α radiation, λ = 0.71073 Å
Hall symbol: -P 2ybc	Cell parameters from 912 reflections
*a* = 9.656 (2) Å	θ = 2.3–26.3°
*b* = 10.617 (2) Å	µ = 2.45 mm^−^^1^
*c* = 11.187 (3) Å	*T* = 298 K
β = 112.874 (4)°	Prism, brown
*V* = 1056.7 (4) Å^3^	0.22 × 0.15 × 0.08 mm
*Z* = 4	

### Data collection

**Table:**

Bruker SMART 1000 CCD diffractometer	2554 independent reflections
Radiation source: fine–focus sealed tube	1758 reflections with 2σ(*I*)
graphite	*R*_int_ = 0.030
ω scans	θ_max_ = 28.3°, θ_min_ = 2.3°
Absorption correction: multi-scan (*SADABS*; Sheldrick, 2004)	*h* = −12→11
*T*_min_ = 0.615, *T*_max_ = 0.828	*k* = 0→14
2554 measured reflections	*l* = 0→14

### Refinement

**Table:**

Refinement on *F*^2^	Primary atom site location: structure-invariant direct methods
Least-squares matrix: full	Secondary atom site location: difference Fourier map
*R*[*F*^2^ > 2σ(*F*^2^)] = 0.034	Hydrogen site location: inferred from neighbouring sites
*wR*(*F*^2^) = 0.076	H-atom parameters constrained
*S* = 1.05	*w* = 1/[σ^2^(*F*_o_^2^) + (0.0327*P*)^2^] where *P* = (*F*_o_^2^ + 2*F*_c_^2^)/3
2554 reflections	(Δ/σ)_max_ = 0.001
128 parameters	Δρ_max_ = 0.33 e Å^−^^3^
0 restraints	Δρ_min_ = −0.34 e Å^−^^3^

### Special details

**Table:**

Geometry. All s.u.'s (except the s.u. in the dihedral angle between two l.s. planes) are estimated using the full covariance matrix. The cell s.u.'s are taken into account individually in the estimation of s.u.'s in distances, angles and torsion angles; correlations between s.u.'s in cell parameters are only used when they are defined by crystal symmetry. An approximate (isotropic) treatment of cell s.u.'s is used for estimating s.u.'s involving l.s. planes.
Refinement. Refinement of *F*^2^ against ALL reflections. The weighted *R*–factor *wR* and goodness of fit *S* are based on *F*^2^, conventional *R*–factors *R* are based on *F*, with *F* set to zero for negative *F*^2^. The threshold expression of *F*^2^ > σ(*F*^2^) is used only for calculating *R*–factors(gt) *etc*. and is not relevant to the choice of reflections for refinement. *R*–factors based on *F*^2^ are statistically about twice as large as those based on *F*, and *R*–factors based on ALL data will be even larger.

### Fractional atomic coordinates and isotropic or equivalent isotropic displacement parameters (Å^2^)

**Table:**

	*x*	*y*	*z*	*U*_iso_*/*U*_eq_	
C1	0.3581 (3)	0.6235 (2)	0.0701 (2)	0.0334 (6)	
C2	0.3833 (3)	0.4480 (2)	0.1999 (3)	0.0426 (7)	
H2A	0.4210	0.4551	0.2936	0.051*	
H2B	0.4086	0.3650	0.1783	0.051*	
C3	0.2123 (3)	0.4657 (3)	0.1417 (3)	0.0504 (8)	
H3A	0.1753	0.4759	0.2103	0.06*	
H3B	0.1639	0.3926	0.0905	0.06*	
C4	0.4210 (3)	0.7245 (2)	0.0176 (2)	0.0340 (6)	
C5	0.3317 (3)	0.8206 (3)	−0.0777 (3)	0.0460 (7)	
H5A	0.3662	0.9032	−0.0445	0.069*	
H5B	0.2275	0.8121	−0.0919	0.069*	
H5C	0.3438	0.8085	−0.1581	0.069*	
C6	0.7922 (3)	0.7897 (2)	0.0918 (3)	0.0385 (6)	
Cl	0.75176 (8)	0.43164 (6)	0.31254 (7)	0.0474 (2)	
N1	0.4522 (2)	0.5445 (2)	0.1476 (2)	0.0361 (5)	
N2	0.5671 (2)	0.71858 (19)	0.0663 (2)	0.0333 (5)	
N3	0.6450 (2)	0.8074 (2)	0.0291 (2)	0.0399 (6)	
N4	0.8837 (3)	0.8692 (2)	0.0669 (2)	0.0552 (7)	
H4A	0.8472	0.9292	0.0121	0.066*	
H4B	0.9795	0.8606	0.1057	0.066*	
Ni	0.65556 (4)	0.58829 (3)	0.18229 (3)	0.03380 (12)	
S1	0.17043 (8)	0.60537 (7)	0.03983 (8)	0.0493 (2)	
S2	0.86881 (8)	0.66908 (7)	0.20402 (7)	0.0465 (2)	

### Atomic displacement parameters (Å^2^)

**Table:**

	*U*^11^	*U*^22^	*U*^33^	*U*^12^	*U*^13^	*U*^23^
C1	0.0295 (14)	0.0359 (14)	0.0342 (15)	−0.0026 (11)	0.0119 (12)	−0.0042 (12)
C2	0.0445 (17)	0.0365 (15)	0.0506 (19)	−0.0033 (13)	0.0225 (15)	0.0017 (13)
C3	0.0422 (17)	0.0473 (17)	0.061 (2)	−0.0105 (14)	0.0187 (16)	0.0021 (15)
C4	0.0331 (15)	0.0317 (14)	0.0340 (15)	0.0010 (11)	0.0094 (12)	−0.0004 (11)
C5	0.0394 (16)	0.0458 (17)	0.0475 (18)	0.0044 (13)	0.0112 (14)	0.0105 (14)
C6	0.0358 (15)	0.0396 (15)	0.0441 (17)	−0.0061 (12)	0.0199 (13)	−0.0028 (13)
Cl	0.0398 (4)	0.0440 (4)	0.0541 (5)	0.0087 (3)	0.0137 (3)	0.0101 (3)
N1	0.0314 (12)	0.0359 (12)	0.0403 (13)	−0.0027 (10)	0.0131 (10)	0.0025 (10)
N2	0.0303 (12)	0.0333 (12)	0.0369 (13)	−0.0006 (9)	0.0137 (10)	0.0004 (10)
N3	0.0393 (13)	0.0383 (13)	0.0458 (14)	−0.0047 (10)	0.0206 (12)	0.0030 (11)
N4	0.0351 (14)	0.0576 (16)	0.0727 (19)	−0.0084 (12)	0.0207 (13)	0.0112 (14)
Ni	0.02818 (19)	0.03349 (19)	0.0382 (2)	0.00139 (15)	0.01121 (15)	0.00176 (15)
S1	0.0281 (4)	0.0485 (5)	0.0661 (5)	−0.0031 (3)	0.0126 (4)	0.0033 (4)
S2	0.0293 (4)	0.0486 (4)	0.0578 (5)	−0.0008 (3)	0.0128 (3)	0.0060 (4)

### Geometric parameters (Å, °)

**Table:**

C1—N1	1.292 (3)	C5—H5B	0.96
C1—C4	1.463 (3)	C5—H5C	0.96
C1—S1	1.720 (3)	C6—N4	1.327 (3)
C2—N1	1.462 (3)	C6—N3	1.332 (3)
C2—C3	1.533 (4)	C6—S2	1.743 (3)
C2—H2A	0.97	Cl—Ni	2.1679 (8)
C2—H2B	0.97	N1—Ni	1.905 (2)
C3—S1	1.817 (3)	N2—N3	1.367 (3)
C3—H3A	0.97	N2—Ni	1.861 (2)
C3—H3B	0.97	N4—H4A	0.86
C4—N2	1.302 (3)	N4—H4B	0.86
C4—C5	1.485 (3)	Ni—S2	2.1554 (9)
C5—H5A	0.96		
			
N1—C1—C4	116.9 (2)	H5B—C5—H5C	109.5
N1—C1—S1	118.2 (2)	N4—C6—N3	117.4 (3)
C4—C1—S1	124.9 (2)	N4—C6—S2	119.2 (2)
N1—C2—C3	109.1 (2)	N3—C6—S2	123.4 (2)
N1—C2—H2A	109.9	C1—N1—C2	114.4 (2)
C3—C2—H2A	109.9	C1—N1—Ni	112.27 (18)
N1—C2—H2B	109.9	C2—N1—Ni	133.07 (18)
C3—C2—H2B	109.9	C4—N2—N3	118.3 (2)
H2A—C2—H2B	108.3	C4—N2—Ni	117.17 (18)
C2—C3—S1	107.85 (19)	N3—N2—Ni	124.56 (16)
C2—C3—H3A	110.1	C6—N3—N2	110.0 (2)
S1—C3—H3A	110.1	C6—N4—H4A	120
C2—C3—H3B	110.1	C6—N4—H4B	120
S1—C3—H3B	110.1	H4A—N4—H4B	120
H3A—C3—H3B	108.4	N2—Ni—N1	83.24 (9)
N2—C4—C1	110.4 (2)	N2—Ni—S2	86.68 (7)
N2—C4—C5	124.5 (2)	N1—Ni—S2	169.68 (7)
C1—C4—C5	125.2 (2)	N2—Ni—Cl	177.76 (7)
C4—C5—H5A	109.5	N1—Ni—Cl	95.06 (7)
C4—C5—H5B	109.5	S2—Ni—Cl	95.08 (3)
H5A—C5—H5B	109.5	C1—S1—C3	90.43 (13)
C4—C5—H5C	109.5	C6—S2—Ni	95.27 (9)
H5A—C5—H5C	109.5		
			
N1—C2—C3—S1	−3.3 (3)	C4—N2—Ni—N1	0.65 (19)
N1—C1—C4—N2	−3.2 (3)	N3—N2—Ni—N1	−179.2 (2)
S1—C1—C4—N2	174.88 (19)	C4—N2—Ni—S2	−177.17 (19)
N1—C1—C4—C5	176.9 (2)	N3—N2—Ni—S2	3.02 (19)
S1—C1—C4—C5	−5.1 (4)	C1—N1—Ni—N2	−2.43 (19)
C4—C1—N1—C2	178.3 (2)	C2—N1—Ni—N2	−175.7 (3)
S1—C1—N1—C2	0.1 (3)	C1—N1—Ni—S2	9.8 (5)
C4—C1—N1—Ni	3.7 (3)	C2—N1—Ni—S2	−163.5 (3)
S1—C1—N1—Ni	−174.50 (12)	C1—N1—Ni—Cl	179.02 (18)
C3—C2—N1—C1	2.2 (3)	C2—N1—Ni—Cl	5.7 (2)
C3—C2—N1—Ni	175.34 (19)	N1—C1—S1—C3	−1.9 (2)
C1—C4—N2—N3	−179.1 (2)	C4—C1—S1—C3	−179.9 (2)
C5—C4—N2—N3	0.8 (4)	C2—C3—S1—C1	2.9 (2)
C1—C4—N2—Ni	1.1 (3)	N4—C6—S2—Ni	−178.7 (2)
C5—C4—N2—Ni	−179.0 (2)	N3—C6—S2—Ni	1.6 (2)
N4—C6—N3—N2	−179.5 (2)	N2—Ni—S2—C6	−2.03 (11)
S2—C6—N3—N2	0.1 (3)	N1—Ni—S2—C6	−14.2 (4)
C4—N2—N3—C6	177.7 (2)	Cl—Ni—S2—C6	176.58 (9)
Ni—N2—N3—C6	−2.5 (3)		

### Hydrogen-bond geometry (Å, °)

**Table:**

*D*—H···*A*	*D*—H	H···*A*	*D*···*A*	*D*—H···*A*
N4—H4B···Cl^i^	0.86	2.51	3.310 (3)	155.
N4—H4A···Cl^ii^	0.86	2.53	3.373 (3)	166.
C3—H3B···S1^iii^	0.97	2.98	3.537 (3)	118.

Symmetry codes: (i) −*x*+2, *y*+1/2, −*z*+1/2; (ii) *x*, −*y*+3/2, *z*−1/2; (iii) −*x*, −*y*+1, −*z*.
